# Influence of EMS-physician presence on survival after out-of-hospital cardiopulmonary resuscitation: systematic review and meta-analysis

**DOI:** 10.1186/s13054-015-1156-6

**Published:** 2016-01-09

**Authors:** Bernd W. Böttiger, Michael Bernhard, Jürgen Knapp, Peter Nagele

**Affiliations:** 1 Department of Anaesthesiology and Intensive Care Medicine, University Hospital of Cologne, Kerpener Str. 62, 50937 Cologne, Germany; 2 Emergency Department, University Hospital of Leipzig, Liebigstr. 20, 04103 Leipzig, Germany; 3 Department of Anesthesiology and Pain Therapy, Bern University Hospital, Freiburgstr. 4, 3010 Bern, Switzerland; 4 Department of Anesthesiology, Washington University School of Medicine, St. Louis, MO USA

**Keywords:** Cardiac arrest, Cardiopulmonary resuscitation, Outcomes, Emergency medical service physicians, Paramedics

## Abstract

**Background:**

Evidence suggests that EMS-physician-guided cardiopulmonary resuscitation (CPR) in out-of-hospital cardiac arrest (OOHCA) may be associated with improved outcomes, yet randomized controlled trials are not available. The goal of this meta-analysis was to determine the association between EMS-physician- *versus* paramedic-guided CPR and survival after OOHCA.

**Methods and Results:**

Studies that compared EMS-physician- *versus* paramedic-guided CPR in OOHCA published until June 2014 were systematically searched in MEDLINE, EMBASE and Cochrane databases. All studies were required to contain survival data. Data on study characteristics, methods, and as well as survival outcomes were extracted. A random-effects model was used for the meta-analysis due to a high degree of heterogeneity among the studies (*I* ^*2*^ = 44 %). Return of spontaneous circulation [ROSC], survival to hospital admission, and survival to hospital discharge were the outcome measures.

Out of 3,385 potentially eligible studies, 14 met the inclusion criteria. In the pooled analysis (n = 126,829), EMS-physician-guided CPR was associated with significantly improved outcomes compared to paramedic-guided CPR: ROSC 36.2 % (95 % confidence interval [CI] 31.0 – 41.7 %) vs. 23.4 % (95 % CI 18.5 – 29.2 %) (pooled odds ratio [OR] 1.89, 95 % CI 1.36 – 2.63, p < 0.001); survival to hospital admission 30.1 % (95 % CI 24.2 – 36.7 %) vs. 19.2 % (95 % CI 12.7 – 28.1 %) (pooled OR 1.78, 95 % CI 0.97 – 3.28, p = 0.06); and survival to discharge 15.1 % (95 % CI 14.6 – 15.7 %) vs. 8.4 % (95 % CI 8.2 – 8.5 %) (pooled OR 2.03, 95 % CI 1.48 – 2.79, p < 0.001).

**Conclusions:**

This systematic review suggests that EMS-physician-guided CPR in out-of-hospital cardiac arrest is associated with improved survival outcomes.

**Electronic supplementary material:**

The online version of this article (doi:10.1186/s13054-015-1156-6) contains supplementary material, which is available to authorized users.

## Background

The optimal emergency medical service (EMS) system configuration and staffing for out-of-hospital cardiopulmonary resuscitation (CPR) are controversial [1–3]. In several countries, EMS physicians are an integral part of prehospital EMS teams and are often dispatched to the most severe cases, including cardiac arrest. EMS physicians have undergone special training in emergency medicine that often goes beyond current advanced cardiac life support standards [1–7]. Despite the intuitive appeal of having EMS physicians guiding out-of-hospital CPR, there is only limited evidence about the influence of EMS-physician-guided CPR on outcomes after out-of-hospital cardiac arrest (OOHCA). Studies comparing the effect of different EMS systems (i.e., EMS-physician-staffed versus nonphysician (paramedic)-staffed systems) and their effects on survival in OOHCA patients are notoriously difficult to conduct and thus are limited [1–3]. Interestingly, almost all large-scale comparative studies demonstrate a survival benefit associated with EMS-physician-guided CPR for OOHCA [2–5, 7].

The goal of this study was therefore to summarize the existing evidence comparing EMS-physician-guided versus paramedic-guided CPR and survival after OOHCA.

## Methods

The Preferred Reporting Items for Systematic reviews and Meta-Analyses (PRISMA) [8] and Meta-analysis Of Observational Studies in Epidemiology (MOOSE) guidelines [9] were followed in this meta-analysis.

### Search strategy

We performed a literature search accessing MEDLINE, EMBASE, and Cochrane databases for studies published until June 2014 using the following search terms and keywords: PubMed: (Heart arrest [mh] OR ((cardiac [tw] OR heart [tw]) AND arrest [tw])) AND (prehospital [tw] OR pre-hospital [tw] OR out-of-hospital [tw] OR “emerg* physician*” [tw] OR “prehosp* physician*” [tw]) AND (ALS [tw] OR advanced card* support* [tw] OR advanced cardiac life support [mh] OR resuscitat* [tw] OR resuscitation [mh] OR cardiopulmonary resuscitation [mh]). The search strategy was based on combinations of Medical Subject Heading terms and text words and was not restricted to a specific language or year of publication. Electronic databases were searched—Cochrane Database for Systematic Reviews and Central Register of Controlled Trials (http://www.cochrane.org/), MEDLINE (http://www.ncbi.nlm.nih.gov/PubMed), and EMBASE (https://www.elsevier.com/solutions/embase-biomedical-research) —and hand searches of journals, review articles, and books were performed. In addition, we manually checked the reference list of each article. The main focus of this study was on prospective clinical trials, and we also included analysis of retrospective observational cohort studies.

### Study selection

Since no randomized controlled clinical trials were available, we included in this meta-analysis all prospective and retrospective observational cohort studies. The following eligibility criteria were required for inclusion: observational cohort studies; comparison between EMS-physician-guided and paramedic-guided CPR; survival data available; adult population; and OOHCA. Articles were considered if published in English or German. For the study by Hagihara et al. [10], we selected only the propensity-matched cohort to reduce selection bias (*n* = 9231 EMS-physician-treated cardiac arrests versus 9231 paramedic-treated cardiac arrests).

### Data extraction

Information about sample size, study design, and characteristics was extracted from the articles as well as the following data: patients treated by EMS physicians and paramedics, patients achieving return of spontaneous circulation (ROSC), surviving to hospital admission, and to hospital discharge, as well as 30-day survival. Survival to hospital discharge was the primary outcome variable. If survival to hospital discharge data were not available, we used ROSC and hospital admission as the primary outcomes. We used 30-day survival data if survival to discharge data were not available.

### Statistical analysis

We performed the analysis with the Comprehensive Meta-Analysis software, version 2.2.064 (Biostat, Englewood, NJ, USA). Risk ratios and 95 % confidence intervals (CIs) were (re)calculated for each study and pooled in both a fixed-effects model and a random effects model. The Comprehensive Meta-Analysis software uses the inverse variance method for weighing studies. However, other methods can be selected, such as Mantel–Haenszel. The results in our meta-analyses did not differ between each method. Heterogeneity among studies was formally assessed by the *Q* and *I* ^2^ statistics. Publication bias was tested with the Egger’s regression test.

## Results

**Fig. 1:**
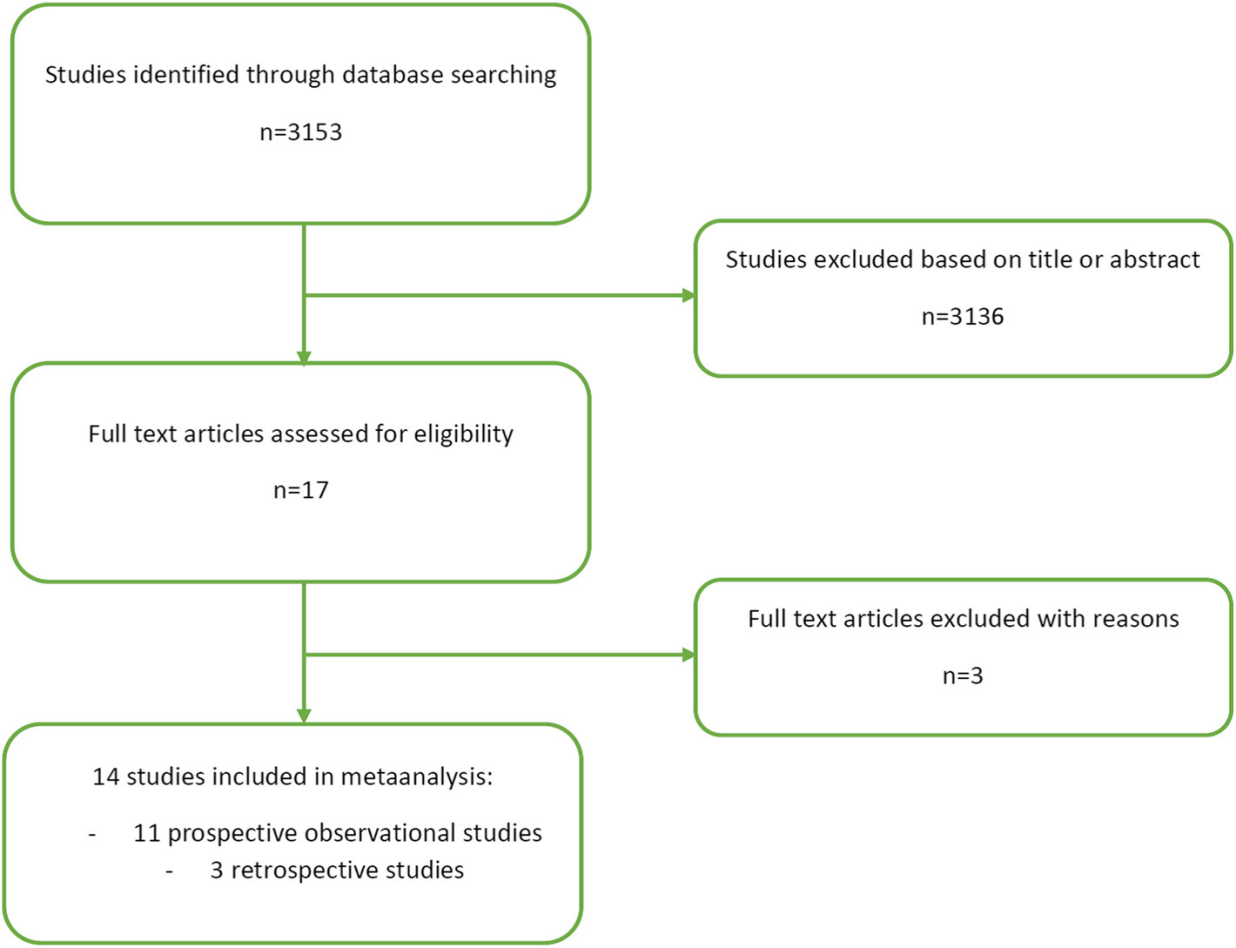
Study selection process (based on PRISMA guidelines)

The literature search identified 3153 publications that met the search criteria. Detailed evaluation of abstracts and full articles resulted in 14 studies that met inclusion and exclusion criteria (Fig. 1, Table 1) [4, 5, 7, 10–20]. Quality of the included studies was variable and the heterogeneity was high (*I* ^2^ = 44 %). The funnel plot of included studies shows a small likelihood of publication bias (Additional file 1: Figure S1). The total pooled sample size was 126,829 cardiac arrest patients.

**Table 1:** Characteristics of included studies with physicians and non-physicians (paramedics) in out-of-hospital CPR

Author	Design	Details	Patients treated by		ROSC		Survival to hospital admission		Survival to hospital discharge		30-day survival	
			Physician (*n*)	Paramedic (*n*)	Physician (*n*/total, %)	Paramedic (*n*/total, %)	Physician (*n*/total, %)	Paramedic (*n*/total, %)	Physician (*n*/total, %)	Paramedic (*n*/total, %)	Physician (*n*/total, %)	Paramedic (*n*/total, %)
Olasveengen et al., 2009 [11]	Retrospective analysis of registry data	2003–2008, contemporaneous, urban, same city, same dispatch criteria	232	741	79/232, 34.0 %	242/741, 32.7 %	With ROSC 66/232, 28.4 %; with ongoing CPR 22/232, 9.5 %; all hospital 88/232, 37.9 %	With ROSC 195/741, 26.3 %; with ongoing CPR 98/741, 13.2 %; all hospital 293/741, 39.5 %	31/232; 13.4 %	78/741, 10.5 %	Not reported	Not reported
Yen et al., 2006 [14]	Prospective, observational multicenter study	1999–2000, contemporaneous, urban, same city, same dispatch criteria	115	43	Not reported	Not reported	17/115, 14.8 %	16/43, 37.2 %	3/115, 2.6 %	4/43, 9.3 %	Not reported	Not reported
Oshige et al., 2005 [15]	Prospective, observational study	2003, contemporaneous, different urban and rural areas: four areas with physician-manned ambulances compared with four areas with paramedic-staffed ambulances, same dispatch criteria	120	222	Not reported	Not reported	49/120, 40.8 %	52/222, 23.4 %	Not reported	Not reported	13/120; 10.8 %	10/222; 4.5 %
Fischer et al., 2003 [4]	Prospective, observational study	1997, contemporaneous, two different cities (city of Bonn, Germany: physician-manned ambulance vs. city of Birmingham, UK: paramedic-staffed ambulances)	918	3380	415/918, 45.2 %	554/3380, 16.4 %	371/918, 40.4 %	362/3380, 10.7 %	135/918, 14.7 %	135/3380, 4.0 %	Not reported	Not reported
Soo et al., 1999 [16]	Retrospective observational study	1991–1994, contemporaneous, same area	70	551	Not reported	Not reported	17/70, 24.3 %	86/551, 15.6 %	11/70, 15.7 %	32/551, 5.8 %	Not reported	Not reported
Kojima et al., 2010 [7]	Prospective, observational study	2005–2008, contemporaneous, propensity score-matched analysis	2072	2072	555/2072, 26.8 %	249/2072, 12.0 %	Not reported	Not reported	Not reported	Not reported	336/2072, 16.2 %	227/2072, 11.0 %
Eisenburger et al., 2001 [12]	Descriptive observational study with prospective data collection	1991–1998, contemporaneous, same rural area	105	13	47/105, 44.8 %	7/13, 53.8 %	Not reported	Not reported	23/105, 21.9 %	3/13, 23.1 %	1-year survival: 20/105, 19.0 %	1-year survival: 1/13, 7.7 %
Dickenson et al., 1997 [13]	Retrospective case review	1994, contemporaneous, same suburban area	9	40	6/9, 66.7 %	12/40, 30.0 %	Not reported	Not reported	4/9, 44.4 %	2/40, 5.0 %	Not reported	Not reported
Hagihara et al., 2014 [10]	Prospective, registry study	2005–2010, contemporaneous, nationwide in Japan, physician not dispatched to the scene but happened to be present during rescue mission for training of the ambulance crew or occasionally when the patient collapsed	9231	9231	2774/9231, 30.1 %	1661/9231, 18.0 %	Not reported	Not reported	Not reported	Not reported	1441/9231, 15.6 %	1169/9231, 12.7 %
Yasunaga et al., 2010 [17]	Prospective, registry study	2005-2007, contemporaneous, nationwide in Japan, in several regions a physician-staffed ambulance is available	without BCPR 1597; with BCPR 1916	without BCPR 53,482; with BCPR 38,077	Not reported not reported	Not reported not reported	Not reported not reported	Not reported not reported	Not reported not reported	Not reported not reported	Without BCPR 185/1597, 11.6 %; with BCPR 287/1916, 15.0 %; all patients: 472/3513, 13.4 %	Without BCPR 3608/53,482, 6.7 %; with BCPR 3642/38,077, 9.6 %; all patients: 7250/91559, 7.9 %
Hampton et al., 1977 [18]	Prospective, interventional study	probably 1975–1976, contemporaneous, same urban area	19	46	Not reported	Not reported	9/19, 47.4 %	8/46, 17.4 %	3/19, 15.8 %	2/46, 4.3 %	Not reported	Not reported
Mitchell et al., 1997 [19]	Prospective, observational study	one calendar year in the middle of the nineties, contemporaneous, 2 different urban areas (Edinburgh, UK: physician-based vs. Milwaukee, USA: paramedic-based)	306	732	116/306, 37.7 %	225/732, 31.1 %	78/306, 25.5 %	159/732, 21.7 %	38/306, 12.4 %	52/732, 7.2 %	Not reported	Not reported
Frandsen et al., 1991 [20]	Prospective, interventional study	paramedic: 1986–1989, physician: 1988, partly contemporaneous, same urban and rural area	85	308	Not reported	Not reported	14/85, 16.5 %	31/308, 10.1 %	11/85, 12.9 %	10/308, 3.2 %	Not reported	Not reported
Fischer et al., 2011 [5]	Prospective, observational study	2001-2004, physician-staffed: urban (Bonn, Germany) and rural (Cantabria, Spain), paramedic-based: urban (Coventry, UK and Richmond, USA)	263	833	89/263, 33.8 %	214/833, 25.7 %	84/263, 31.9 %	110/833, 13.2 %	Not reported	Not reported	Not reported	Not reported

*CPR* cardiopulmonary resuscitation, *ROSC* return of spontaneous circulation, *BCPR *bystander cardiopulmonary resuscitation

**Fig. 2:**
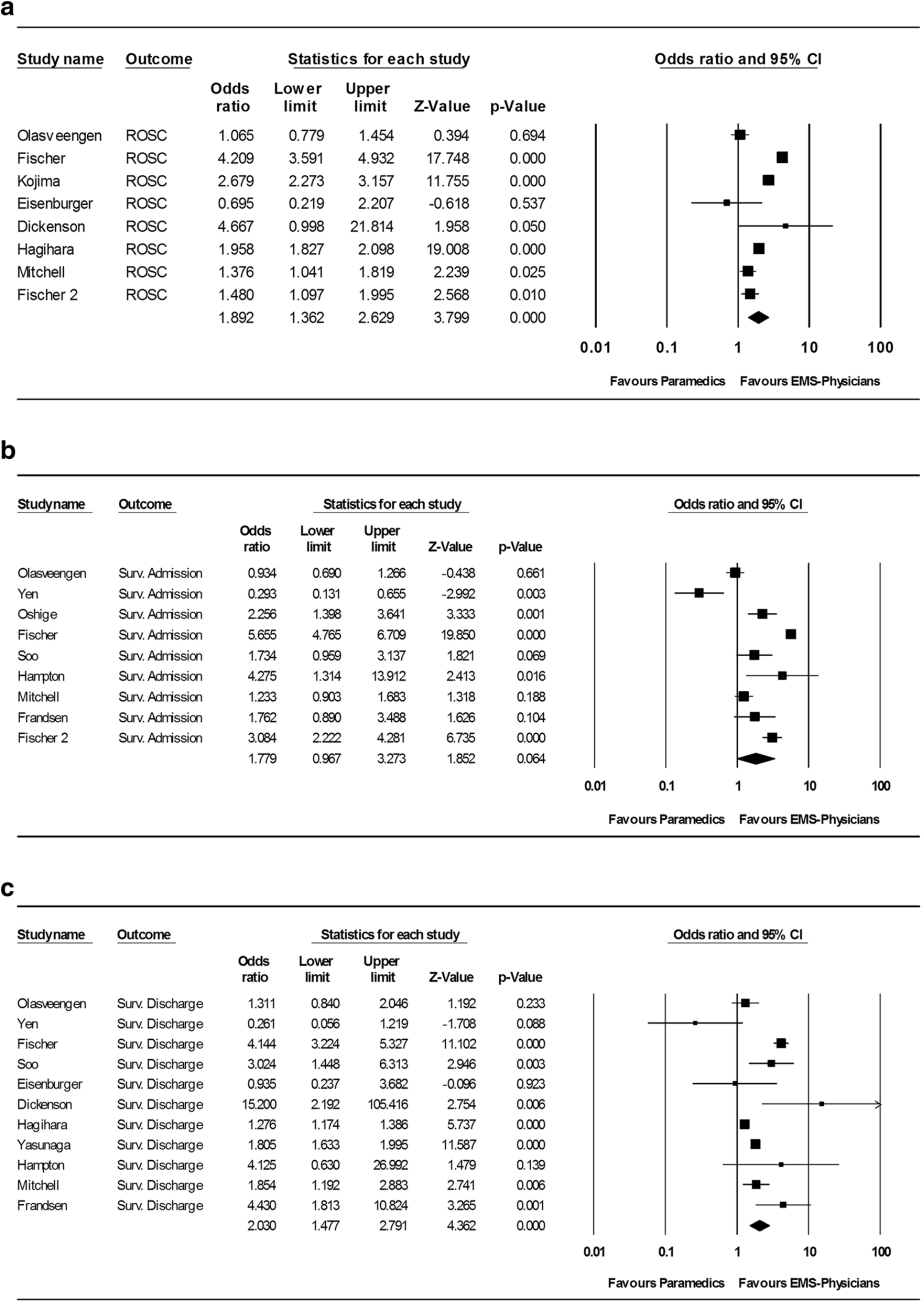
Outcomes after CPR comparing EMS-physician-guided CPR with paramedic-guided CPR. **a** Return of spontaneous circulation (*ROSC*). **b** Survival to hospital admission. **c** Survival to hospital discharge. *CI* confidence interval, *EMS* emergency medical services, *Surv.* survival

In the pooled analysis, EMS-physician-guided CPR was associated with significantly improved outcomes compared with paramedic-guided CPR. The pooled estimate for ROSC for EMS-physician-guided CPR was 36.2 % (95 % CI 31.0–41.7 %) and for paramedics was 23.4 % (95 % CI 18.5–29.2 %) (pooled odds ratio (OR) 1.89, 95 % CI 1.36–2.63, *p* <0.001) (Fig. 2a; Additional file 1: Figure S2A). The pooled estimated survival-to-hospital admission rate for EMS-physician-guided CPR was 30.1 % (95 % CI 24.2–36.7 %) and for paramedics was 19.2 % (95 % CI 12.7–28.1 %) (pooled OR 1.78, 95 % CI 0.97–3.28, *p* = 0.06; Fig. 2b; Additional file 1: Figure S2B). The pooled estimated survival-to-hospital discharge rate for EMS-physician-guided CPR was 15.1 % (95 % CI 14.6–15.7 %) and for paramedics was 8.4 % (95 % CI 8.2–8.5 %) (pooled OR 2.03, 95 % CI 1.48–2.79, *p* <0.001; Fig. 2c; Additional file 1: Figure S2C).

## Discussion

The results of this meta-analysis show that CPR guided by EMS physicians is associated with improved rates of ROSC, hospital admission, and hospital discharge compared with CPR guided by paramedics in OOHCA patients.

This meta-analysis included 14 international studies with a pooled sample size of more than 126,000 patients. Two studies from Japan [10, 17] accounted for nearly 90 % of the total sample size and thus had the biggest weight in the meta-analysis. Because the individual studies were largely consistent in the effect size estimate, we did not perform sensitivity analyses excluding these two studies.

This study excluded several studies that had excellent methodology but did not directly compare EMS-physician-guided with paramedic-guided CPR, which may influence its generalizability. In several studies, EMS physicians provided advanced life support whereas paramedics were only allowed to perform basic life support without the administration of resuscitation drugs or advanced airway management. On the other hand, most countries that have a paramedic-only EMS system allow paramedics a nearly identical scope of prehospital practice compared with EMS physicians. Therefore, it is unclear whether our results show predominantly the superiority of advanced life support in OOHCA over basic life support or a true superiority of EMS-physician-guided CPR. In the multicenter Ontario Prehospital Advanced Life Support Study (OPLAS) study, Stiell et al. [21] directly compared advanced with basic life support for OOHCA and found no positive effect of advanced life support by paramedics on survival after OOHCA. This observation would argue against a predominant effect of advanced life support over basic life support.

This meta-analysis has several limitations. First, meta-analyses pool existing evidence and are thus dependent on the scientific quality of included studies. Typically, meta-analyses of randomized controlled trials provide the strongest and most robust evidence. In our study, no randomized controlled trials exist that compare EMS-physician-guided with paramedic-guided CPR and probably never will, due to the fact that whole states and countries operate one particular EMS system and switching systems is very costly. Despite the nonrandomized nature of studies included in this meta-analysis [4, 5, 7, 10–20], the evidence favoring EMS-physician-guided CPR for OOHCA appears to be robust since almost all studies found a similarly positive survival effect. Second, selection bias may have influenced individual study results. In some EMS systems, EMS-physician-staffed ambulances may have not been dispatched to cases of OOHCA that were futile based on the assessment of an ambulance crew on the scene. Alternatively, EMS physicians may have determined on scene that initiation of CPR was not appropriate, which may have influenced the denominator of “potential cardiac arrests”. This would have limited EMS-physician-guided CPR to OOHCA cases with a higher likelihood of successful resuscitation. Third, the geographic distribution of EMS systems is highly variable and is often influenced by many historical factors that all may have confounded the results of this meta-analysis.

If the results of this meta-analysis are true—that is, EMS-physician-guided CPR provides survival benefit in OOHCA over paramedic-guided CPR—what may be the causes? What could EMS physicians provide beyond what paramedics already contribute? First, it has been demonstrated that because of the limited number of invasive procedures performed by EMS crews (like airway management, tracheal intubation, etc.) in out-of-hospital patients, it is very difficult to obtain or maintain life-saving skills [22–25]. As an example, even after 150 attempts at intubating the trachea in elective surgical patients under optimal conditions in the operating room the success rate is only 95 % [26]. In the out-of-hospital setting, however, conditions are generally more difficult, leading to more challenging prehospital airway management [27, 28]. On the other hand, EMS physicians are often anesthesiologists who maintain airway skills in the operating room while working only part-time in EMS medicine. Second, physician presence during CPR has been reported to increase compliance with guidelines, resulting in less hands-off time during CPR [11].

A randomized controlled trial comparing EMS-physician-guided versus paramedic-guided CPR will not be possible due to many reasons. Therefore, despite the significant limitations which are readily acknowledged, this systematic review provides the only available evidence for the effectiveness of a paramedic versus EMS-physician-based emergency response system for prehospital cardiac arrest. Perhaps there may be opportunities for natural experiments when EMS systems change from paramedics to EMS physicians or vice versa. Additional analyses using large-scale registry data may help to elucidate this topic in the future.

## Conclusions

In summary, findings from this meta-analysis suggest that CPR guided by EMS physicians is associated with improved survival compared with paramedic-guided CPR in OOHCA patients.

## Additional file

Additional file 1:
**Figure S1 showing the funnel plot for publication bias analysis and Figure S2 showing the pooled event rates for ROSC, survival to hospital admission, and survival to hospital discharge. **(DOCX 95 kb)

## Abbreviations

CI: Confidence interval
CPR: Cardiopulmonary resuscitation
EMS: Emergency medical service
MOOSE: Meta-analysis Of Observational Studies in Epidemiology
OOHCA: Out-of-hospital cardiac arrest
OR: Odds ratio
PRISMA: Preferred Reporting Items for Systematic reviews and Meta-Analyses
ROSC: Return of spontaneous circulation
