# Supplementary material for: Influence of EMS-physician presence on survival after out-of-hospital cardiopulmonary resuscitation: systematic review and meta-analysis
Source: Crit Care. 2016 Jan 9;20:4. doi: 10.1186/s13054-015-1156-6 (PMC4706668; doi:10.1186/s13054-015-1156-6)
Supplement: Additional file 1: — Figure S1 showing the funnel plot for publication bias analysis and Figure S2 showing the pooled event rates for ROSC, survival to hospital admission, and survival to hospital discharge. (DOCX 95 kb) [file 13054_2015_1156_MOESM1_ESM.docx]

**Supplemental Figure 1: Funnel Plot for Publication Bias Analysis**

**Supplemental Figure 2. Pooled Event Rates for ROSC, Survival to Hospital Admission and Survival to Hospital Discharge.**

These figures provide pooled event rate estimates, e.g. an event rate of ROSC of 0.194 = 19.4% rate of ROSC. All estimates are from random-effects models.

Figure S2A. ROSC

Figure S2B. Survival to Hospital Admission

Figure S2C. Survival to Hospital Discharge
